# Pre-Clinical Validation of A Novel Continuous Intra-Abdominal Pressure Measurement Equipment (SERENNO)

**DOI:** 10.3390/life12081161

**Published:** 2022-07-30

**Authors:** Salar Tayebi, Robert Wise, Ali Pourkazemi, Johan Stiens, Manu L. N. G. Malbrain

**Affiliations:** 1 Department of Electronics and Informatics, Vrije Universiteit Brussel, 1050 Brussels, Belgium; salar.tayebi@vub.be (S.T.); apourkaz@etrovub.be (A.P.); jstiens@etrovub.be (J.S.); 2 Adult Intensive Care, John Radcliffe Hospital, Oxford University Hospitals Trust, Oxford OX3 7LE, UK; rob.wise@ouh.nhs.uk; 3 Discipline of Anaesthesia and Critical Care, School of Clinical Medicine, University of KwaZulu-Natal, Durban 4000, South Africa; 4 First Department of Anaesthesiology and Intensive Therapy, Medical University Lublin, 20-954 Lublin, Poland; 5 Medical Data Management, Medaman, 2440 Geel, Belgium; 6 International Fluid Academy, 3360 Lovenjoel, Belgium

**Keywords:** intra-abdominal pressure, abdominal compartment, continuous measurement, pre-clinical validation

## Abstract

**Introduction:** Increased intra-abdominal pressure (IAP) has an important impact on morbidity and mortality in critically ill patients. The SERENNO Sentinel system (Serenno Medical, Yokne’am Illit, Israel) is a novel device that allows automatic and continuous IAP measurements. **Aims:** Pre-clinical validation in a bench model study comparing the new device with the gold standard method and two other continuous IAP measurement devices. **Methods:** IAP measurement with the novel SERENNO device (IAP_SER_) was compared with the gold standard IAP_H2O_ (water column height) and two other automatic and continuous IAP measurement devices: IAP_CiM_ measured via the CiMON device (Pulsion Medical Systems, Munich, Germany) and IAP_SPIE_ measured using the Spiegelberg device (Spiegelberg, Hamburg, Germany), which previously received the CE mark for clinical applications. The IAP measurement was performed six times (*n* = 6) at each pressure value (between 0 and 35 mmHg) with different methods and the height of the water column in a bench-top phantom was used as the reference IAP for further interpretations. In addition to the quadruple comparisons, intra- and inter-observer variability of IAP measurements were also calculated. Correlation studies and Bland and Altman’s analyses were performed in addition to the concordance study. **Results:** The CiMON and Spiegelberg devices showed a greater dynamic range and standard deviation when recording IAP_CiM_ and IAP_SPIE_ compared with IAP_SER_. In general, the maximum and minimum values of IAP recorded with each device (at each level of IAP_H2O_) were significantly different from each other. However, the average values were in very good agreement. The highest correlation was observed between IAP_SER_ and IAP_H2O_, and IAP_SER_ and IAP_SPIE_ (R = 0.99, *p* = 0.001 for both comparisons and intra- and inter-observer measurements). Although the CiMON and SERENNO systems were in very good agreement with each other, a slightly smaller correlation coefficient was found between them (R = 0.95, *p* = 0.001, and R = 0.96, *p* = 0.001 for intra- and inter-observer measurements, respectively). When compared to the gold standard (IAP_H2O_), Bland and Altman’s analysis showed a mean difference of +0.44, −0.25, and −0.04 mmHg for the intra-observer measurements and +0.18, −0.75, and −0.58 mmHg for the inter-observer measurements for IAP_SER_, IAP_CiM_, and IAP_SPIE_, respectively. IAP_SER_ showed a small positive bias (overestimation), while IAP_CiM_ and IAP_SPIE_ showed a negative bias (underestimation) when compared to IAP_H2O_. Further statistical analysis showed a concordance coefficient of 100% with an excellent ability of the SERENNO system in tracking IAP_H2O_ changes. **Conclusions:** Pre-clinical validation of a new IAP monitoring device (SERENNO) showed very promising results when compared with the gold standard and other continuous techniques; however, clinical trials should be followed as the next stage of the validation process. Based on the actual research guidelines, the SERENNO system can be used interchangeably with the gold standard.

## 1. Introduction

Intra-abdominal pressure (IAP) is an important physiological parameter in critically ill patients that represents the steady-state pressure within the abdominal compartment. IAP values higher than 12 mmHg result in lower cardiac preload, which in turn, results in lower cardiac output, and reduced perfusion pressure to the distal abdominal organs, finally leading to multiple organ dysfunction and failure depending on the severity of intra-abdominal hypertension (IAH) [1,2,3]. Moreover, late detection of IAH can potentially result in abdominal compartment syndrome (ACS), which is a more severe and potentially lethal condition with sustained IAP values higher than 20 mmHg and new-onset organ failure [3]. Previous investigations in critically ill patients showed an IAH prevalence of more than 50% during the first week of intensive care unit (ICU) admission [4,5,6]. Although some studies show a lower incidence of ACS (approximately 3–5%) in general ICU patients [7], the risk of ACS in critically ill patients should not be underestimated. For instance, a recent study that included 138 post-cardiac surgery patients showed an IAH occurrence rate of 100% during the first 24 h post-operatively, with a sustained IAP pressure above 20 mmHg for approximately 5 h [8]. Therefore, continuous IAP monitoring is of great importance to prevent late detection of IAH, development of ACS, and potentially contribute to a reduced length of ICU stay. In addition to patient care, shorter ICU stays would significantly reduce hospitalization costs for patients and healthcare providers.

Although several measurement techniques have been proposed [3,9,10,11,12,13,14], IAP measurement via the bladder is the only approved gold standard method by the abdominal compartment society (formerly known as the World Society of Abdominal Compartment Syndrome, WSACS). Intra-bladder pressure measurement should be performed in the supine position after instilling a maximum of 25 mL of saline into the bladder, with the zero-reference level where the mid-axillary line crosses the iliac crest [1]. Since the abdominal compartment is primarily fluid in character and, thus, follows Pascal’s law, pressure is equally transmitted and IAP can be estimated by measurement of the pressure inside a hollow organ contained within this cavity (i.e., bladder, stomach, rectum, uterus, etc.).

In the present study, the SERENNO sentinel system (Serenno Medical, Yokne’am Illit, Israel) is a novel automatic and continuous IAP measurement device that is connected to an existing Foley catheter, hence avoiding extra manipulations, costs, and risk of infection. The system also measures urine output (UO), but this was not the purpose of this study. By using an abdominal phantom, this study aimed to perform a preclinical validation comparing the IAP values obtained with the new device to the gold standard (water column height) and two other existing (already CE-marked) continuous IAP monitoring devices using a balloon-tipped nasogastric probe, namely the CiMON (Pulsion Medical Systems, Munich, Germany) and the Spiegelberg device (Spiegelberg, Hamburg, Germany).

## 2. Materials and Methods

### 2.1. SERENNO System

**Figure 1:**
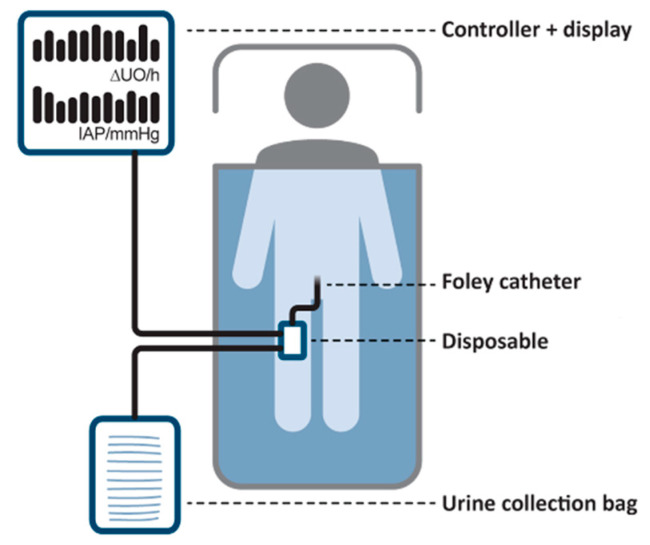
A schematic representation of the SERENNO system. This system consists of two main parts; the disposable and the controller unit as well as standard hospital equipment, namely the Foley catheter and the urine collection bag.

A general schematic representation of the SERENNO system is shown in Figure 1. The disposable is a special pressure-sensing fluid pump that is connected in series between the Foley catheter and the urine collection bag. The disposable is then connected with air tubes to the controller, and together they form the main components of the system.

**Figure 2:**
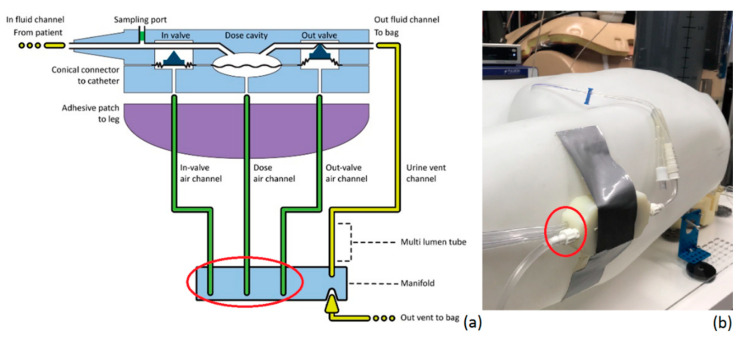
Detailed representation of (**a**) the disposable and (**b**) the connection of the disposable on the human abdominal phantom at the level of the artificial bladder (zero reference). The red circle indicates the three air tubes that connect the disposable to the controller.

As illustrated in Figure 2, it has two urine ports, one being the in-fluid channel, which is connected to the Foley catheter. The second one is the out-fluid channel, which connects the disposable to the urine collection bag. In addition to the in- and out-fluid channels, it has three chambers that are connected to the controller unit. The in-valve chamber is an input gate that can block or allow urine to flow into the disposable by adjusting the air pressure inside the in-valve air channel. The out-valve chamber is completely identical to the in-valve chamber; however, it controls the urine flow going out of the disposable. Lastly, the dose chamber is a fixed volume chamber with a membrane that can freely move by adjusting the air pressure inside the dose air channel to fill or empty the dosing chamber with 1 mL of fluid.

**Figure 3:**
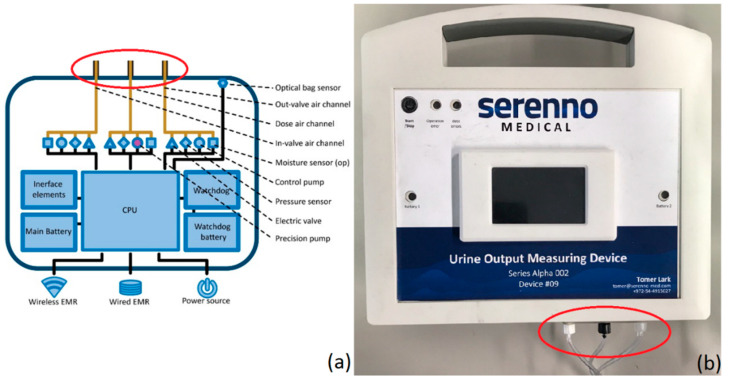
Detailed schematic representation of the controller unit (**a**) and picture of the actual controller (**b**). The red circle indicates the three air tubes that connect the disposable to the controller.

The second main part of the SERENNO system is the controller unit, which is presented in Figure 3. The controller adjusts the air pressure inside the three air channels (in, out, and dosing) by a set of pneumatic-electrical components (the air valve, pressure sensor, and electric pump for each channel). A watchdog circuit component is designed to maintain function in case of power loss or CPU failure.

The controller operates and collects data from the disposable unit by measuring and controlling the air pressure in each of the three air channels in a sequential procedure to measure both IAP and urine output. The system operates in cycles: each cycle allows 1 mL of urine (or fluid in the bench-top model) to move from the patient (or artificial bladder) to the collection bag, based on the pressure presented from the bladder. The IAP monitoring is done automatically in combination with the urine output monitoring (the UO monitoring process will not be detailed here). During each cycle, the device halts all pressure manipulations in a specific state where the bladder is partially filled and there is a fluid column creating pressure that is directly applied to the membrane in the dosing cavity. This membrane is designed to allow accurate and reliable transfer of this pressure from the fluid pressure on one side of the membrane to air pressure on the other side.

An air pressure sensor that is present in the controller unit is then put in direct contact with the air pressure in the dosing cavity (below the membrane). That sensor continuously monitors the dose chamber air pressure, but the pressure is calculated as IAP only during a specific time in the process.

When there is a need to measure IAP, the system automatically aligns the different valves and pressure settings to have a steady-state and lasting connection between the bladder (by way of the fluid column), the membrane (by way of transferring the fluid pressure to air pressure), the tubing (while accounting for expected time/friction delays), and finally the sensor. The collected waveform signal is then processed to locate the end-expiratory pressure of several respiratory cycles and ignores the noise and other artifacts.

### 2.2. Spiegelberg System

**Figure 4:**
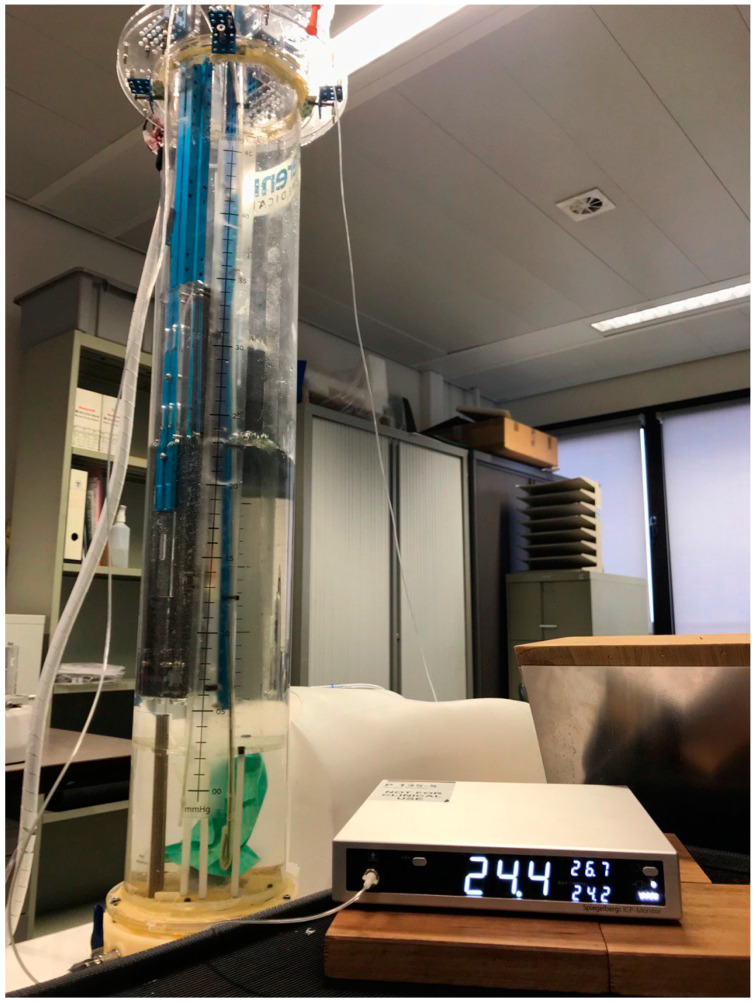
The Spiegelberg measurement device (Spiegelberg GmbH, Hamburg, Germany) showing the mean, maximal, and minimal IAP values (in mmHg).

The Spiegelberg (Spiegelberg GmbH, Hamburg, Germany) measurement device (see Figure 4) has been described previously and consists of a nasogastric tube-like catheter (outer diameter 3 mm) equipped with an air-filled balloon (total filling volume 0.1 mL) connected to a device for automatic zeroing, control, and pressure measurement [15]. A Spiegelberg-catheter is introduced in the human abdominal phantom at the mid-level of the artificial bladder (see further).

### 2.3. CiMON System

**Figure 5:**
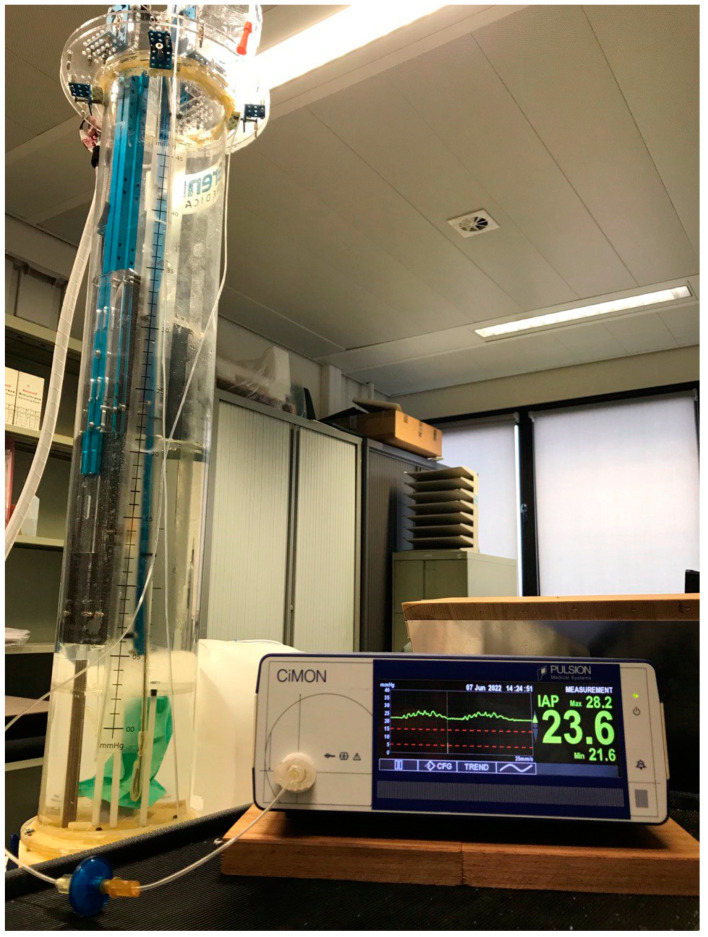
The CiMON measurement device (Pulsion Medical Systems, Munich, Germany) showing a graphical representation of actual IAP-tracing with respiratory variations and heartbeat artifacts on the left-hand side and mean, maximal, and minimal IAP (in mmHg) on the right.

The CiMON system (Pulsion Medical Systems, Munich, Germany) consists of a nasogastric probe (outer diameter 5.3 mm) with a small inflatable balloon (total filling volume 1.1 mL of air) located at the distal tip of the probe (see Figure 5). The probe has one lumen that connects the air-filled balloon with the IAP monitor and one feeding lumen that can also be used for introducing a guidewire. The balloon is connected to a device for automatic zeroing, control, and pressure measurement [16]. A CiMON-catheter was positioned in the human abdominal phantom at the mid-level of the artificial bladder (see further).

### 2.4. Human Phantom

**Figure 6:**
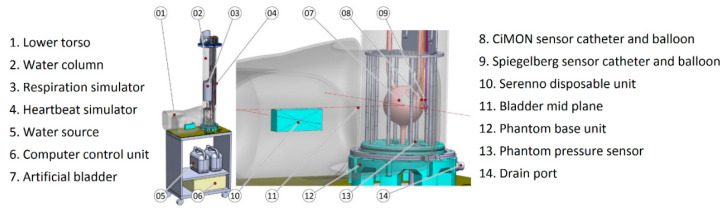
A human bench-top phantom was used to validate the SERENNO system against the gold standard and the Spiegelberg and CiMON devices.

A human abdominal phantom was designed by the research group for this study and used to validate the SERENNO system against the gold standard (water column height) and the two IAP measurement devices. As illustrated in Figure 6, the phantom consists of a computer-controlled water column to simulate different IAP levels (0, 5, 10, 15, 20, 25, 30, and 35 mmHg). At the bottom of the water column, there is a green balloon that represents the bladder containing a Foley catheter and filled with fluid that simulates urine. The hydrostatic pressure on the bladder (IAP_H2O_) was controlled by adjusting the height of the water column above the simulated bladder. The heartbeat and the respiratory cycles were simulated by having two moving components inside the water tank. The large cylinder in the middle of the water column simulated the impact of respiration on IAP with an inspiratory increase and an expiratory decrease. When the cylinder moved inside the water column, it increased the water column height, which in turn, resulted in a higher hydrostatic pressure exerted on the artificial bladder. The other moving component simulated the heartbeat and applied slight vibration and pressure fluctuations in the water column by periodic water column height increases and reductions.

**Figure 7:**
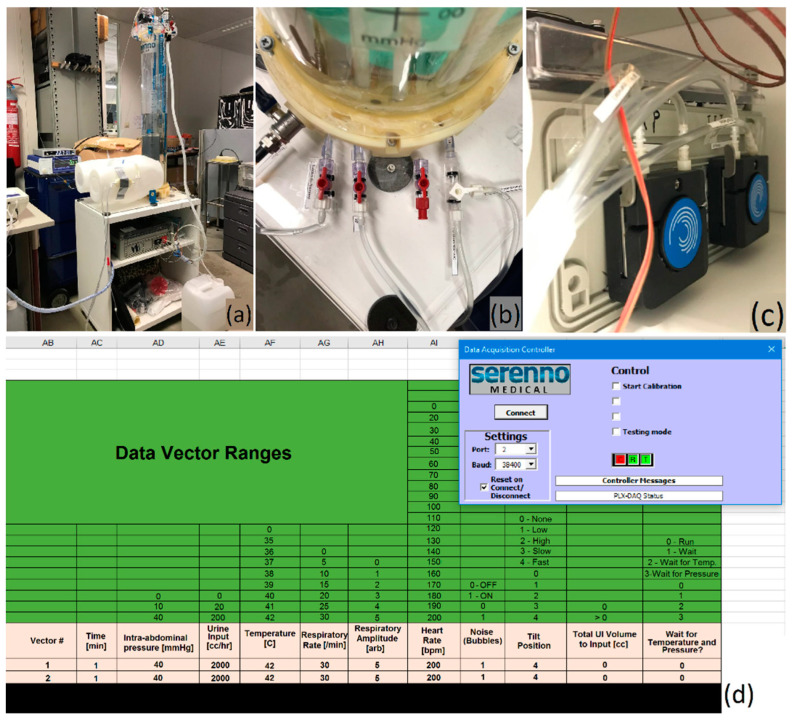
The human phantom was used in this study to validate the SERENNO system against the gold standard (water column height) and the Spiegelberg and CiMON devices. (**a**) A general representation of the phantom set-up. (**b**) The in-and output ports of the phantom adjust the water column height and allow the instillation of fluid into the artificial bladder inside the phantom. (**c**) The two fluid pumps instill or remove fluid from the water tank and artificial bladder. (**d**) The user interface of the phantom. As can be seen, several parameters including the IAP level, heart rate, respiration rate, respiratory excursions, etc. can be pre-defined.

The investigator could change the respiratory rate and heartbeats per minute via a computer connected to a controller, and this could be adjusted to simulate fast and slow and deep and shallow breathing, and heartbeat intensity (see Figure 7).

For the purpose of this study, the respiratory rate was set at 20 breaths per minute (with amplification of 5 mmHg, representing the IAP change during the respiratory cycle) and the heart rate at 100 beats per minute (bpm). The CiMON and Spiegelberg devices operated via a balloon-tipped catheter that was positioned at the mid-plane level of the artificial bladder, and the SERENNO disposable unit was similarly positioned at the end of a Foley catheter inserted into the lower torso at the same height.

### 2.5. Validation Study Set-Up

**Figure 8:**
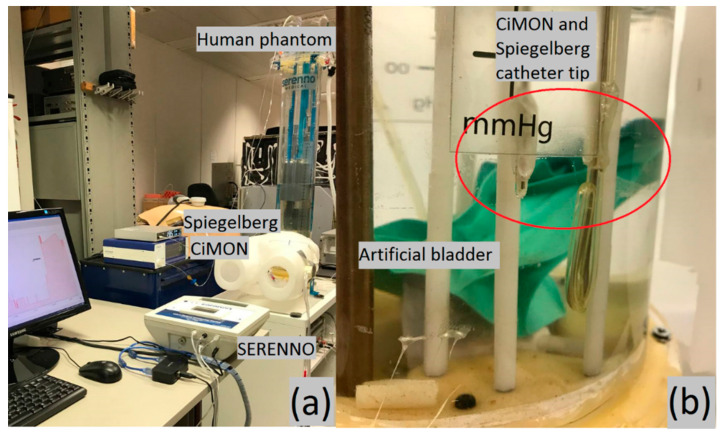
The measurement set-up. (**a**) The phantom, CiMON, Spiegelberg, and SERENNO systems. (**b**) The artificial bladder in relation to the catheter tips of the CiMON (**right**) and Spiegelberg (**left** balloon) systems.

The disposable of the SERENNO system was connected to the phantom’s bladder via a Foley catheter. The pressure probes of the CiMON and Spiegelberg catheters were placed at the same height as the artificial bladder inside the phantom (see Figure 8). Subsequently, heart rate, respiration rate, and respiratory excursions were adjusted to 100 bpm, 20 respirations per minute, and 5 mmHg, respectively. Different IAP values between 0 and 35 mmHg were simulated inside the phantom by increasing the height of the water column inside the tank. The maximum, minimum, and mean IAP values (mimicking the inspiratory and expiratory variations during each respiratory cycle) were repeatedly (*n* = 6) measured by the same observer at each pre-set IAP value (height of water column) with the SERENNO system to identify intra-observer variability. The same IAP-derived parameters were also recorded blindly using the CiMON and Spiegelberg devices. To assess inter-observer variability, the measurements were repeated by three observers. Due to the structural restrictions of the phantom, it was not possible to apply any heartbeat/respiration simulation at 0 and 5 mmHg IAP_H2O_.

### 2.6. Statistical Analysis

Results of continuous data that followed a Gaussian distribution are presented as a mean with standard deviations (±SD), unless otherwise stated. Mean values were compared using a student’s *t*-test. Paired IAP measurements between the new device and the gold standard, or by the other two different IAP methods, were compared statistically using different methods. First, we used Pearson correlation, Lin’s concordance correlation, and linear regression analysis. Two methods were considered equal if the line of identity crosses the origin of the *X* and *Y*-axis and if R² (R = Pearson’s correlation coefficient) was greater than 0.6. Additionally, the Lin’s concordance correlation coefficient (R_c_) was used as a measure to show how well one system can reproduce the measurements of another system. Two methods can reproduce their data perfectly or substantially if the R_c_ is higher than 0.99 or between 0.95 and 0.99, respectively. Subsequently, the intra-class correlation coefficient (ICC) was also calculated between the measurement systems to check the reliability of the measurements. Secondly, we calculated bias, which is defined as the mean difference between IAP_H2O_ and IAP_SER_. Subsequently, precision and limits of agreement (LA) were defined as the standard deviation of bias and bias ± 1.96 precision according to Bland and Altman [17]. Thirdly, the percentage error was calculated by multiplying precision into two and dividing the result by the mean IAP values of that technique. Finally, the ability of IAP_SER_ to track changes or trends in IAP_H2O_ was assessed by plotting ΔIAP_SER_ against ΔIAP_H2O_ during the same time interval (four quadrants trend plot). The concordance coefficient was then calculated as the percentage of pairs with the same direction of change after exclusion of pairs with both a ΔIAP_SER_ and ΔIAP_H2O_ ≤ 2.5 mmHg (or less than 15% of change) and exclusion of pairs with either ΔIAP_SER_ or ΔIAP_H2O_ equal to zero [18]. Statistical analysis was performed with Excel (Microsoft Excel 2007, Microsoft Corporation, Redmond, WA, USA) and SPSS statistical package for the social sciences (SPSS Inc., Chicago, IL, USA). A *p*-value smaller than 0.05 was considered significant.

## 3. Results

### 3.1. Dynamic Respiratory IAP Variations

**Figure 9:**
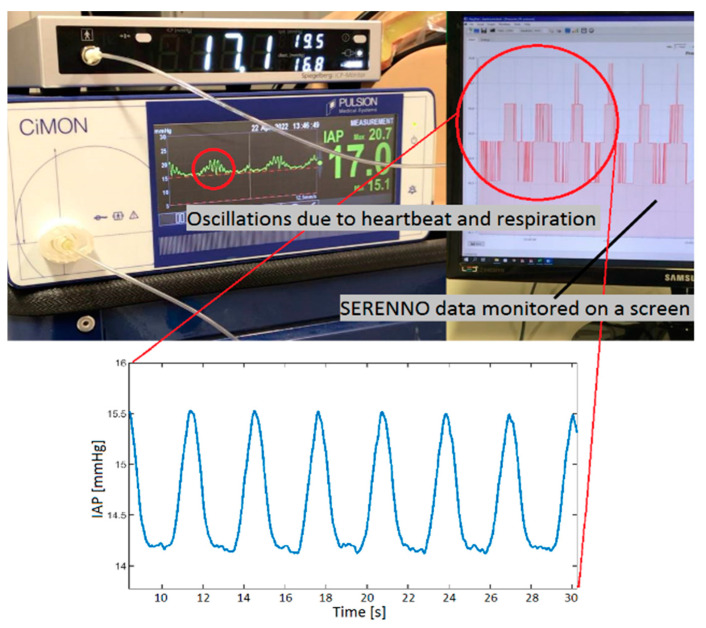
An image of the CiMON, Spiegelberg, and SERENNO systems measuring the intra-abdominal pressure (IAP) at a pre-set water column height of 15 mmHg. Two oscillations with different frequencies can be seen on the CiMON and SERENNO signals, which are due to the simulated heartbeat (higher frequency) and respiration (lower frequency).

Figure 9 shows the CiMON, Spiegelberg, and SERENNO systems measuring the IAP at 15 mmHg. As can be seen, oscillations with different frequencies exist in the (raw) signals, which represent the heartbeat (higher frequency) and respiratory cycle (lower frequency).

The recorded IAP values (mean ± standard deviation) for each device are presented in Table 1, providing an overview of the intra- and inter-observer variabilities.

The mean and standard deviation of the IAP measurements via CiMON, Spiegelberg, SERENNO, and the water tank are represented in Figure S1. At each pre-set IAP value (water column height), three different values are reported for each measurement method, representing the minimum, average, and maximum IAP recorded by each device. The CiMON and Spiegelberg measurements showed a larger dynamic range in IAP values (due to respiratory cycle), as compared to the SERENNO results. Although the dynamic range of the methods used is slightly different from each other, their average values are in agreement. Concerning the coefficient of variation for each device, SERENNO showed a smaller variation in most of the recorded IAP values (both intra- and inter-observer measurements).

### 3.2. IAP Correlations

**Figure 10:**
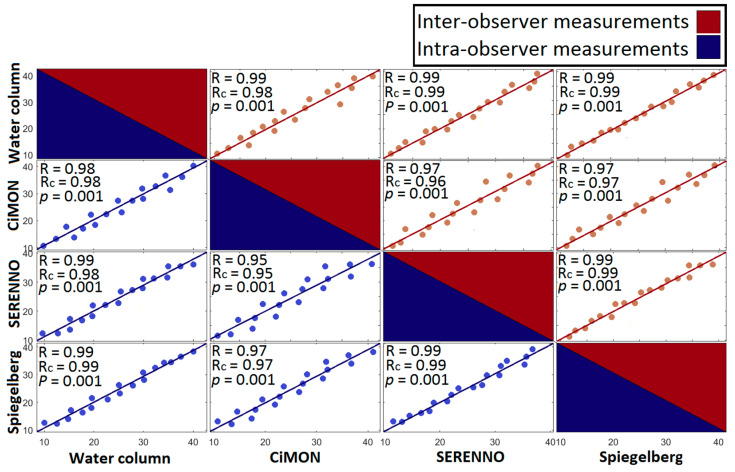
The Pearson (R) and Lin’s concordance (R_c_) correlation matrix of the intra-observer and inter-observer measurements were obtained by performing a correlation analysis between each pair of measurement methods.

We observed a very good correlation and concordance coefficients between all paired measurement comparisons. As can be seen in Figure 10, the highest correlation was observed between IAP_SER_ and IAP_H2O_ and IAP_SER_ and IAP_SPIE_ results (R = 0.99, *p* = 0.001). In contrast, the correlation between IAP_SER_ and IAP_CiM_ had the least (but still excellent) strength (R = 0.95, *p* = 0.001 and R = 0.96, *p* = 0.001 for intra- and inter-observer measurements, respectively).

Taking the R^2^ into account, we can see that the values are between 0.90 (the least R^2^ between IAP_SER_-IAP_CiM_) and 0.98 (the highest R^2^ between IAP_SER_-IAP_SPIE_ and IAP_SER_-IAP_H2O_). Good agreement can be seen between the intra- and inter-observer variabilities. The highest concordance correlation coefficient was observed between IAP_SER_ and IAP_H2O_ and IAP_SER_ and IAP_SPIE_ results (R_c_ = 0.99), and a relatively lower concordance was seen between IAP_SER_ and IAP_CiM_ (R_c_ = 0.95 and R_c_ = 0.96 for the intra- and inter-observer measurements). Moreover, a relatively high intra-class correlation coefficient of 0.984 and 0.982 was obtained between different measurement systems for inter- and intra-observer measurements, respectively (see Figure S2).

### 3.3. Bland and Altman’s Analysis and Percentage Error

**Figure 11:**
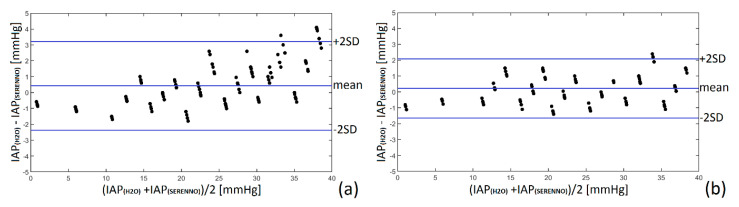
Bland and Altman’s analysis for the SERENNO system versus water column for (**a**) the intra-observer and (**b**) inter-observer measurements. SERENNO system revealed a positive mean difference (underestimation) compared with the IAP of the water column. The small values of bias, precision, and limits of agreement confirm excellent interchangeability between the SERENNO system and the gold standard.

To have a better understanding of the interchangeability of the measurement results, Bland and Altman’s analysis was performed between the reference IAP value (water column height) and the other measurement methods, as previously described [17]. Therefore, the mean difference between the recorded IAP values with each device and the gold standard (water column height) was calculated as bias. Subsequently, having the precision of the recorded data (the standard deviation), the upper and lower limits of agreement were defined as bias + 1.96 precision. All the results are shown in Table 2 and Figure 11 and Figure S3 in detail.

As tabulated, the bias value for all the techniques (either inter- or intra-observer measurements) was less than 1 mmHg. The Spiegelberg device (intra-observer) and the SERENNO device (inter-observer) had the smallest bias and LA when compared to the gold standard. On average, the Spiegelberg, CiMON, and SERENNO showed a percentage error of 7.89%, 14.67%, and 11.74, respectively.

### 3.4. Concordance Analysis

The ability to keep track of dynamic changes in IAP was assessed using concordance plots. ΔIAP_H2O_ and ΔIAP_SER_ are plotted against each other in Figure S4. The exclusion area was defined as the region with both ΔIAP_H2O_ and ΔIAP_SER_ smaller than 2.5 mmHg or the ΔIAP_H2O_ or ΔIAP_SER_ equal to zero. The SERENNO system showed excellent ability in tracking ΔIAP_H2O_ fluctuations.

On average, ΔIAP_H2O_ during IAP elevation was 5.00 ± 0.41 mmHg, while the ΔIAP_SER_ was 4.58 ± 0.46 mmHg. In contrast, during IAP reduction, ΔIAP_H2O_ was −5.00 ± 0.41 and ΔIAP_SER_ was −4.70 ± 0.40 mmHg. In general, the SERENNO system showed an excellent ability in tracking ΔIAP_H2O_ fluctuations (concordance coefficient of 100%); however, its ability in tracking the changes during IAP reduction was more robust compared with the same IAP fluctuations during IAP elevation.

## 4. Discussion

In the present validation study, the SERENNO sentinel system is tested as a novel IAP measurement device. The IAP_SER_ was compared to the gold standard (IAP_H2O_) and two existing automatic and continuous methods (IAP_CiM_ and IAP_SPIE_), which are already CE-marked for clinical applications. We used a simulation phantom capable of artificially creating various IAP levels as needed as well as artificial respiration and cardiac pulsation artifacts.

The CiMON and Spiegelberg showed a greater dynamic range and standard deviation in recording IAP compared with SERENNO, which presented better agreement compared to the phantom pressure sensor. In general, the maximum and minimum values of each device (at each IAP) were significantly different from each other. However, the average values were in very good agreement. Bland and Altman’s results showed a mean difference of −0.25, +0.44, and −0.04 mmHg for the intra-observer measurements and −0.75, +0.18, and −0.58 mmHg for the inter-observer measurements for the CiMON, SERENNO, and Spiegelberg, respectively. Although a positive mean difference was seen for the SERENNO device, the CiMON and Spiegelberg showed a negative mean of difference. In other words, SERENNO showed systematically higher IAP values compared with the water column, while CiMON and Spiegelberg showed a systematically smaller IAP compared to the water column. These differences might be generated due to a slight height difference between the sensor location in each measuring system. Further statistical analysis showed a significant correlation between all the measurement devices. The highest correlation was observed between the SERENNO-water tank and SERENNO-Spiegelberg results (R = 0.99, *p* = 0.001). Although the CiMON and SERENNO systems were in very good agreement with each other, a slightly smaller correlation coefficient was seen between them (R = 0.95, *p* = 0.001 and R = 0.96, *p* = 0.001 for intra- and inter-observer measurements, respectively). Moreover, a concordance correlation coefficient of 0.99 between the SERENNO-Spiegelberg and SERENNO-water column shows the robustness of the SERENNO system to reproduce the measurements of the Spiegelberg and gold standard methods. Although the R_c_ between the SERENNO and CiMON systems was 0.95, it still shows a considerable reproducibility between these two systems.

Based on the WSACS recommendations of research and validation criteria for a novel IAP method, the bias should be less than 1 mmHg with a precision and limit of agreements less than 2 and 4 mmHg to allow two techniques to be used interchangeably [19]. Taking Bland and Altman’s results into account, we can see that all the bias values (either for intra- or inter-observer measurements) were less than 1 mmHg. Additionally, the measurement precision, defined as the standard deviation of the bias, was smaller than 2 mmHg. Additionally, limits of agreement were smaller than 4 mmHg for all the systems. As illustrated in Table 2, the percentage error of the studied systems was less than 25%, which is compatible with the research guidelines. Moreover, the correlation analyses performed between different methods showed a *p*-value less than 0.05, which was assumed as significant.

## 5. Limitations

Although our phantom mimicked respiratory variations and heartbeat simulations very well, it is still far from reality. The impact of patient movements, posture, presence of pelvic tumors, etc. should be investigated by clinical investigations.

## 6. Conclusions

The SERENNO device showed excellent results when compared to the gold standard (using the water column height) and two other existing automatic and continuous IAP measurement techniques (using a balloon-tipped nasogastric catheter). According to the WSACS guidelines, the SERENNO, CiMON, and Spiegelberg systems can be used interchangeably as the bias, precision, and limits of agreement were less than 1, 2, and 4 mmHg, respectively. However, further clinical investigations should be performed to validate the SERENNO systems for clinical applications.

## Figures and Tables

**Table 1:** Mean, standard deviation, and coefficient of variation (CV) of the recorded IAP values with different devices during intra- and inter-observer measurements as compared to the gold standard (pre-set height water column).

IAP_H2O_	IAP_CiM_		IAP_SER_		IAP_SPIE_	
Gold Standard [mmHg]	Intra- Observer [mmHg]	Inter- Observer [mmHg]	Intra- Observer [mmHg]	Inter- Observer [mmHg]	Intra- Observer [mmHg]	Inter- Observer [mmHg]
**0**	0.3 ± 0.0 CV = 0%	1.5 ± 0.1 CV = 6.6%	0.9 ± 0.0 CV = 0%	1.0 ± 0.0 CV = 0%	0.8 ± 0.0 CV = 0%	1.1 ± 0.0 CV = 0%
**5**	5.4 ± 0.1 CV = 1.8%	6.7 ± 0.0 CV = 0%	6.0 ± 0.0 CV = 0%	5.7 ± 0.1 CV = 1.7%	5.8 ± 0.0 CV = 0%	6.2 ± 0.1 CV = 1.6%
**10**	13.2 ± 0.1 CV = 0.7%	12.5 ± 0.4 CV = 3.2%	12.9 ± 0.1 CV = 0.7%	12.6 ± 0.2 CV = 1.6%	12.7 ± 0.1 CV = 0.8%	12.7 ± 0.4 CV = 3.4%
**15**	17.8 ± 0.2 CV = 1.1%	17.7 ± 0.3 CV = 1.7%	17.6 ± 0.2 CV = 1.4%	17.8 ± 0.3 CV = 1.7%	17.2 ±0.1 CV = 0.6%	17.9 ± 0.2 CV = 1.1%
**20**	22.7 ± 0.3 CV = 1.3%	22.0 ± 0.2 CV = 1.4%	22.3 ± 0.3 CV = 1.3%	22.2 ± 0.2 CV = 0.9%	22.1 ± 0.4 CV = 1.8%	22.3 ± 0.2 CV = 1.7%
**25**	27.3 ± 0.2 CV = 0.7%	27.6 ± 0.1 CV = 0.4%	27.3 ± 0.3 CV = 1.1%	27.1 ± 0.1 CV = 0.4%	26.8 ± 0.3 CV = 1.1%	27.4 ± 0.1 CV = 0.4%
**30**	32.3 ± 0.4 CV = 1.2%	32.2 ± 0.2 CV = 0.6%	31.2 ± 0.2 CV = 0.6%	31.8 ± 0.2 CV = 0.6%	32.0 ± 0.4 CV = 1.3%	32.1 ± 0.1 CV = 0.3%
**35**	36.6 ± 0.2 CV = 0.5%	37.3 ± 0.2 CV = 0.5%	35.8 ± 0.3 CV = 0.8%	36.7 ± 0.1 CV = 0.3%	36.3 ± 0.1 CV = 0.3%	37.2 ± 0.1 CV = 0.3%

**Table 2:** Results of Bland and Altman’s analysis comparing the different study devices to the gold standard (water column height) with respect to intra- and inter-observer variability. Results are expressed in mmHg.

*Study* *Method*	Mean IAP [mmHg]	Bias (Difference) [mmHg]	Precision (SD) [mmHg]	LLA [mmHg]	ULA [mmHg]	PE [%]
		**Intra-observer variability**				
*CiMON*	18.66	−0.25	1.28	−2.76	+2.26	13.71
*SERENNO*	19.44	+0.34	1.39	−2.39	+3.06	14.30
*Spiegelberg*	19.39	−0.04	0.87	−1.74	+1.67	8.97
		**Inter-observer variability**				
*CiMON*	19.70	−0.75	1.54	−3.76	+2.26	15.63
*SERENNO*	20.40	+0.12	0.94	−1.72	+1.96	9.21
*Spiegelberg*	19.64	−0.58	0.67	−1.89	+0.73	6.82

SD: standard deviation, LLA: lower limit of agreement, ULA: upper limit of agreement, PE: percentage error.

## Data Availability

Derived data supporting the findings of this study in addition to the processing algorithms are available from the corresponding author on request.

## Supplementary Materials

The following supporting information can be downloaded at: https://www.mdpi.com/article/10.3390/life12081161/s1, Figure S1: Mean and standard deviation of the IAP measurements via CiMON, Spiegelberg, and SERENNO systems; Figure S2: The intra-class correlation graph; Figure S3: Bland and Altman’s analysis results for CiMON, and Spiegelberg systems; Figure S4: Concordance plot showing the ability of the SERENNO system to track the dynamic changes in the reference IAP.
