# Supplementary material for: Pre-Clinical Validation of A Novel Continuous Intra-Abdominal Pressure Measurement Equipment (SERENNO)
Source: Life (Basel). 2022 Jul 30;12(8):1161. doi: 10.3390/life12081161 (PMC9410406; doi:10.3390/life12081161)
Supplement: Supplementary file 1 [file life-12-01161-s001.zip › life-1784425-supplementary.pdf]

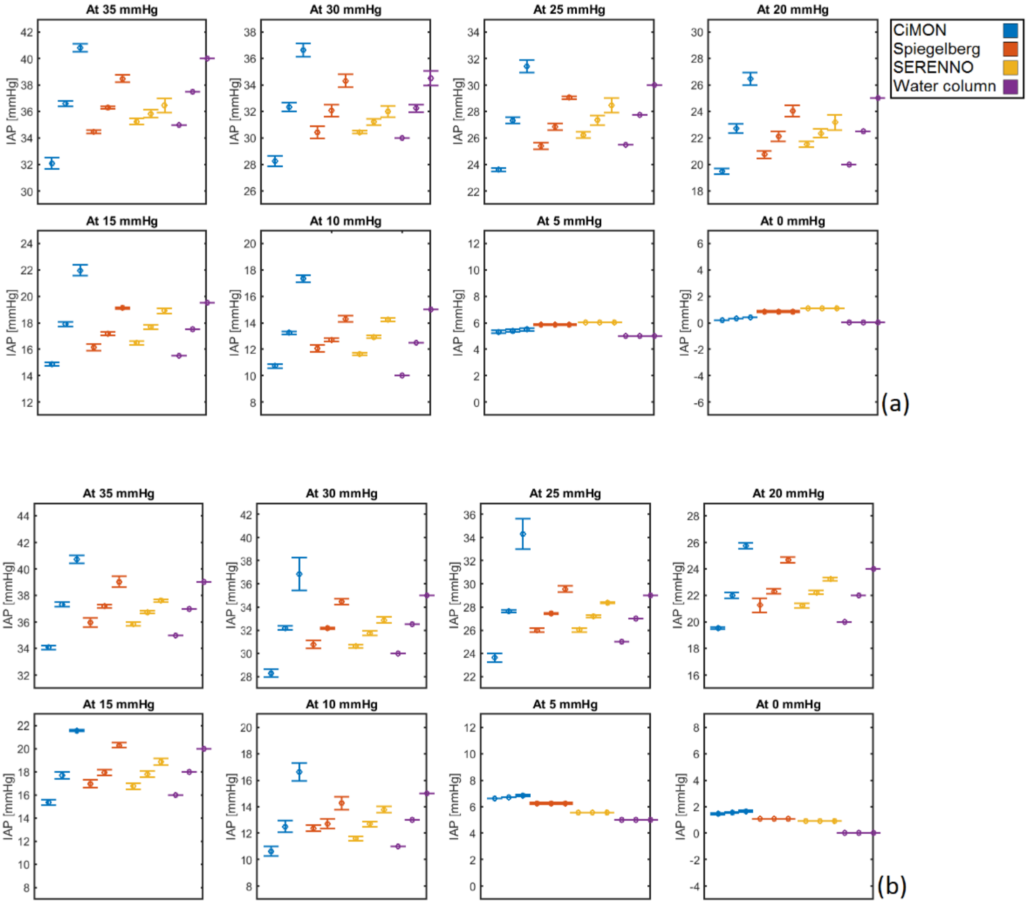

Figure S1. Mean and standard deviation of the IAP measurements via CiMON, Spiegelberg, and SERENNO systems. (a) Intra-observer variation of repeatedly (n = 6) recorded intra-abdominal pressure (IAP) values (in mmHg) obtained with the different study devices. At each pre-set IAP level (water column height), the mean and standard deviation is shown for the minimum (end-expiratory), average, and maximum (end-inspiratory) measured IAP (b). Inter-observer variation.

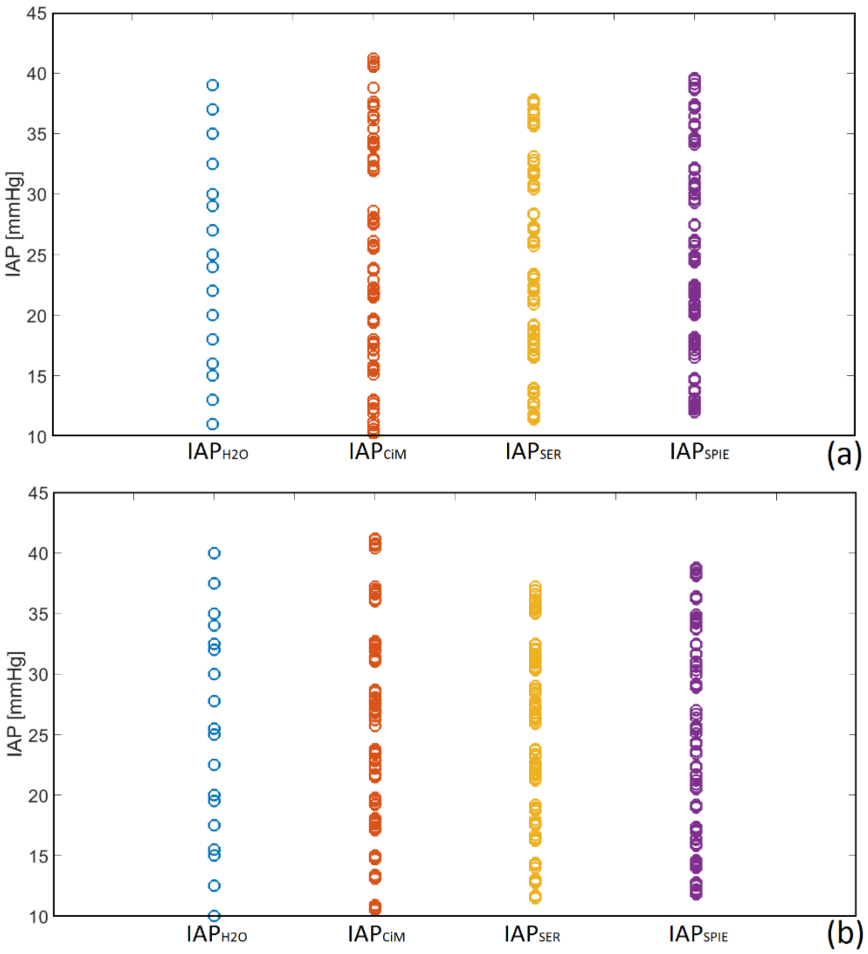

Figure S2. The intra-class correlation graph for (a) the inter-observer and (b) intra-observer measurements. A relatively high intra-class correlation coefficient (ICC) (0.984 and 0.982 for inter- and intra-observer measurements, respectively) was obtained between different measurement systems, which shows the high reliability of the measurements.

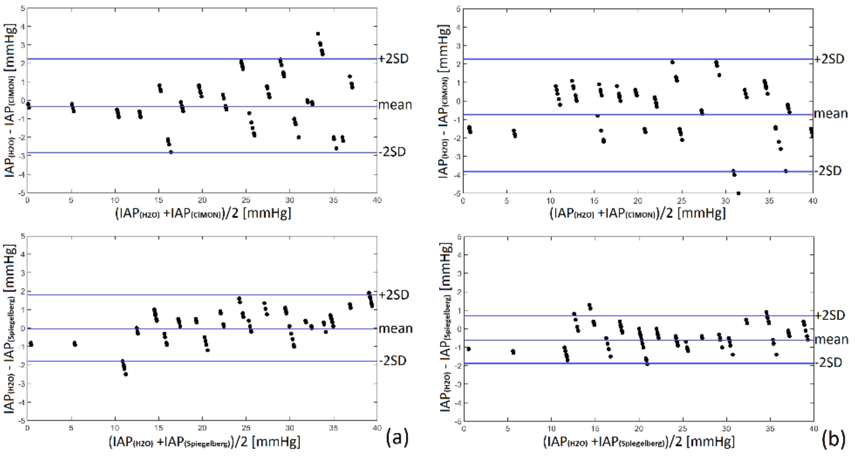

Figure S3. Bland and Altman's analysis results for (a) the intra-observer and (b) inter-observer measurements. In contrast to the SERENNO system, the Spiegelberg and CiMON devices showed a negative mean difference (overestimation). The bias, precision and limits of agreement were small and

confirm excellent interchangeability between the different techniques and the gold standard.

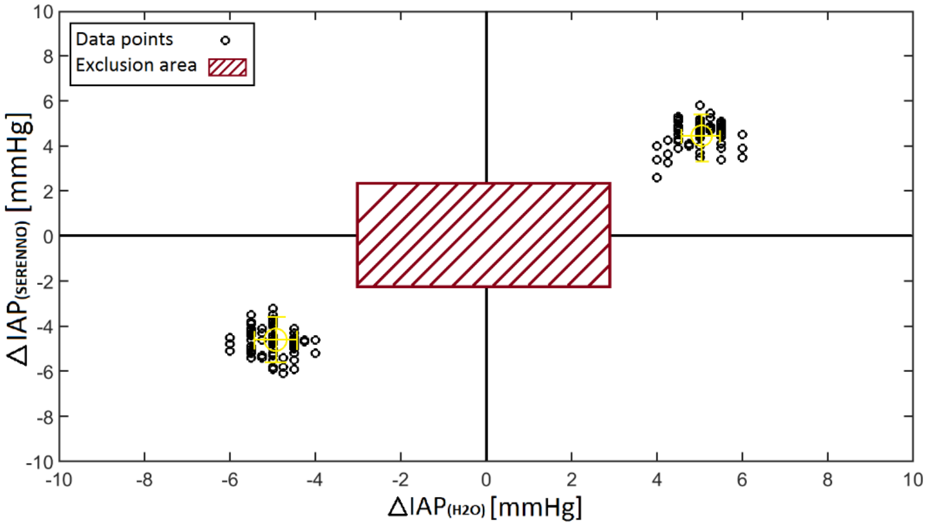

Figure S4. Concordance plot showing the ability of the SERENNO system to track the dynamic changes in the reference IAP. Consecutive ΔIAP_{H2O} and ΔIAP_{SER} were calculated and plotted in relation to each other. The exclusion area is defined as the region where ΔIAP_{H2O} and ΔIAP_{SER} are both smaller than 2.5 mmHg or one of them is 0.
